# Supplementary material for: A fault-intrusion-tolerant system and deadline-aware algorithm for scheduling scientific workflow in the cloud
Source: PeerJ Comput Sci. 2021 Nov 2;7:e747. doi: 10.7717/peerj-cs.747 (PMC8576554; doi:10.7717/peerj-cs.747)
Supplement: Supplemental Information 6 [file peerj-cs-07-747-s006.zip › Myproject1.1/docs/org/workflowsim/Job.html]

Job


JavaScript is disabled on your browser.


- Overview
- Package
- Class
- Use
- Tree
- Deprecated
- Index
- Help

- Prev Class
- Next Class

- Frames
- No Frames

- All Classes

- Summary:
- Nested |
- Field |
- Constr |
- Method

- Detail:
- Field |
- Constr |
- Method


org.workflowsim

## Class Job

- java.lang.Object
- - org.cloudbus.cloudsim.Cloudlet
  - - org.workflowsim.Task
    - - org.workflowsim.Job

- ---

    

  ```
  public class Job
  extends Task
  ```

  Job is an extention to Task. It is basically a group of tasks. In
  WorkflowSim, the ClusteringEngine merges tasks into jobs (group of tasks) and
  the overall runtime of a job is the sum of the task runtime.

  Since:
  :   WorkflowSim Toolkit 1.0

- - ### Field Summary

    - ### Fields inherited from class org.cloudbus.cloudsim.Cloudlet

      `accumulatedBwCost, CANCELED, costPerBw, CREATED, FAILED, FAILED_RESOURCE_UNAVAILABLE, INEXEC, PAUSED, QUEUED, READY, RESUMED, SUCCESS, vmId`
  - ### Constructor Summary

    Constructors

    Constructor and Description

    `Job(int jobId,
    long jobLength)`

    Allocates a new Job object.
  - ### Method Summary

    Methods

    | Modifier and Type | Method and Description |
    | --- | --- |
    | `void` | `addTaskList(java.util.List list)` Adds a task list to the existing task list |
    | `java.util.List` | `getParentList()` Gets the list of the parent tasks and override its super function |
    | `java.util.List<Task>` | `getTaskList()` Gets the list of tasks in this job |
    | `void` | `setTaskList(java.util.List list)` Sets the list of the tasks |

    - ### Methods inherited from class org.workflowsim.Task

      `addChild, addChildList, addFile, addParent, addParentList, getChildList, getDepth, getFileList, getImpact, getPriority, getType, setChildList, setDepth, setFileList, setImpact, setParentList, setPriority, setType`
    - ### Methods inherited from class org.cloudbus.cloudsim.Cloudlet

      `addRequiredFile, deleteRequiredFile, getActualCPUTime, getActualCPUTime, getAllResourceId, getAllResourceName, getClassType, getCloudletFileSize, getCloudletFinishedSoFar, getCloudletFinishedSoFar, getCloudletHistory, getCloudletId, getCloudletLength, getCloudletOutputSize, getCloudletStatus, getCloudletStatusString, getCloudletTotalLength, getCostPerSec, getCostPerSec, getExecStartTime, getFinishTime, getNetServiceLevel, getNumberOfPes, getProcessingCost, getRequiredFiles, getReservationId, getResourceById, getResourceId, getResourceName, getStatus, getStatusString, getSubmissionTime, getSubmissionTime, getUserId, getUtilizationModelBw, getUtilizationModelCpu, getUtilizationModelRam, getUtilizationOfBw, getUtilizationOfCpu, getUtilizationOfRam, getVmId, getWaitingTime, getWallClockTime, getWallClockTime, hasReserved, isFinished, requiresFiles, setClassType, setCloudletFinishedSoFar, setCloudletLength, setCloudletStatus, setExecParam, setExecStartTime, setNetServiceLevel, setNumberOfPes, setRequiredFiles, setReservationId, setResourceParameter, setResourceParameter, setSubmissionTime, setUserId, setUtilizationModelBw, setUtilizationModelCpu, setUtilizationModelRam, setVmId, write`
    - ### Methods inherited from class java.lang.Object

      `clone, equals, finalize, getClass, hashCode, notify, notifyAll, toString, wait, wait, wait`

- - ### Constructor Detail


    - #### Job

      ```
      public Job(int jobId,
         long jobLength)
      ```

      Allocates a new Job object. The job length should be greater than or
      equal to 1.

      Parameters:
      :   `jobId` - the unique ID of this job
      :   `jobLength` - the length or size (in MI) of this task to be executed
          in a PowerDatacenter
  - ### Method Detail


    - #### getTaskList

      ```
      public java.util.List<Task> getTaskList()
      ```

      Gets the list of tasks in this job

      Returns:
      :   the list of the tasks


    - #### setTaskList

      ```
      public void setTaskList(java.util.List list)
      ```

      Sets the list of the tasks

      Parameters:
      :   `list,` - list of the tasks


    - #### addTaskList

      ```
      public void addTaskList(java.util.List list)
      ```

      Adds a task list to the existing task list

      Parameters:
      :   `list,` - task list to be added


    - #### getParentList

      ```
      public java.util.List getParentList()
      ```

      Gets the list of the parent tasks and override its super function

      **Overrides:**
      :   `getParentList` in class `Task`

      Returns:
      :   the list of the parents


- Overview
- Package
- Class
- Use
- Tree
- Deprecated
- Index
- Help

- Prev Class
- Next Class

- Frames
- No Frames

- All Classes

- Summary:
- Nested |
- Field |
- Constr |
- Method

- Detail:
- Field |
- Constr |
- Method
